# Childhood morphea does not impair self-perception

**DOI:** 10.1186/1546-0096-9-S1-P78

**Published:** 2011-09-14

**Authors:** R Carlomagno, G Russo, C Forni, F Orlando, R Vitale, N Scotti, M Alessio

**Affiliations:** 1 Department of Pediatrics,Federico II University, Naples, Italy

## Background

Morphea (localized scleroderma) is an uncommon chronic disease that occurs primarily in children, limited to skin. Morphea influences virtually child's life, causing lifelong dysfunction through localized growth failure and joint contracture.

## Aim

To ascertain whether morphea affects perception of quality of life.

## Methods

We studied 19 patients attending our Department (68,4% female, median age 13,6 yrs). The Childhood Health Assessment Questionnaire (CHAQ) was administered to all patients. The CHAQ is a disease specific instrument selected because of its widespread use in the pediatric rheumatology literature and ease of administration. The CHAQ measures functional ability in eight activities of daily living: dressing and grooming, arising, eating, walking, hygiene, reach, grip and activities. The items with the highest score in a domain determine the score for that domain. These eight domains are then averaged into a summary score called the disability index which may range from 0 to 3, with the higher scores meaning higher disability. The CHAQ also provided an assessment of discomfort using a 10 cm VAS visual analogical scale (VAS) for the evaluation of overall wellbeing.

## Results

The median CHAQ score was 0,43 (range 0-3). The median VAS score was 1,5. Despite the potentially disfiguring effects of morphea, our subjects had normal self-perception.

## Conclusions

This finding runs contrary to hypothesis that morphea would result in a lower self-esteem. Our findings suggest that pediatric morphea, like many other chronic childhood diseases, does not lead to significant reductions in self-worth.
